# Precision treatment of Singleton Merten syndrome with ruxolitinib: a case report

**DOI:** 10.1186/s12969-022-00686-7

**Published:** 2022-04-11

**Authors:** Philip Broser, Ursula von Mengershausen, Katrin Heldt, Deborah Bartholdi, Dominique Braun, Christine Wolf, Min Ae Lee-Kirsch

**Affiliations:** 1 Department of Pediatric Neurology, Children’s Hospital of Eastern Switzerland, Sankt Gallen, Switzerland; 2 Department of Pediatric Endocrinology, Children’s Hospital of Eastern Switzerland, Sankt Gallen, Switzerland; 3 Department of Human Genetics, University Hospital Bern, Bern, Switzerland; 4 Department of Pediatrics, Medizinische Fakultät Carl Gustav Carus, Technische Universität Dresden, Dresden, Germany

**Keywords:** Singleton Merten syndrome, Type I interferon, Therapy, Janus kinase inhibitor, Ruxolitinib, Auto inflammation, Autoimmunity

## Abstract

**Background:**

Singleton-Merten syndrome 1 (SGMRT1) is a rare type I interferonopathy caused by heterozygous mutations in the *IFIH1* gene. *IFIH1* encodes the pattern recognition receptor MDA5 which senses viral dsRNA and activates antiviral type I interferon (IFN) signaling. In SGMRT1, *IFIH1* mutations confer a gain-of-function which causes overactivation of type I interferon (IFN) signaling leading to autoinflammation.

**Case presentation:**

We report the case of a nine year old child who initially presented with a slowly progressive decline of gross motor skill development and muscular weakness. At the age of five years, he developed osteoporosis, acro-osteolysis, alveolar bone loss and severe psoriasis. Whole exome sequencing revealed a pathogenic de novo* IFIH1* mutation, confirming the diagnosis of SGMRT1. Consistent with constitutive type I interferon activation, patient blood cells exhibited a strong IFN signature as shown by marked up-regulation of IFN-stimulated genes. The patient was started on the Janus kinase (JAK) inhibitor, ruxolitinib, which inhibits signaling at the IFN-α/β receptor. Within days of treatment, psoriatic skin lesions resolved completely and the IFN signature normalized. Therapeutic efficacy was sustained and over the course muscular weakness, osteopenia and growth also improved.

**Conclusions:**

JAK inhibition represents a valuable therapeutic option for patients with SGMRT1. Our findings also highlight the potential of a patient-tailored therapeutic approach based on pathogenetic insight.

## Background

Heterozygous gain-of-function mutations in the *IFIH1* gene underlie a spectrum of autoinflammatory phenotypes including Aicardi–Goutières syndrome type 7 (AGS7) [1, 2] and Singleton-Merten syndrome type 1 (SGMRT1) [3]. *IFIH1* encodes interferon-induced helicase C domain-containing protein 1, also known as melanoma differentiation associated gene 5 protein (MDA5), a pattern recognition receptor of the innate immune system which plays a pivotal role in antiviral defense. IFIH1/MDA5 recognizes viral double-stranded RNA (dsRNA) in the cytosol and upon ligand binding activates antiviral type I interferon-(IFN) signaling [4]. *IFIH1* mutations in AGS7 and SGMRT1 act as gain-of-function mutations that lead to inappropriate sensing of self-derived RNA, resulting in constitutive overproduction of type I IFN with subsequent autoinflammation [1, 2]. As such, AGS and SGMRT are also referred to as type I Interferonopathies, a genetically and phenotypically heterogenous group of autoinflammatory and autoimmune diseases associated with perturbation of the type I IFN system [5]. While AGS is characterized by inflammatory neurodegeneration and skin disease, the clinical features of SGMRT comprise abnormal calcification of the aorta and cardiac valves, alveolar bone loss, dental caries, osteoporosis, psoriasis, and muscular weakness [3]. However, a phenotypic overlap between these disorders has been described, suggesting that AGS and SGMRT due to *IFIH1* gain-of-function mutations constitute facets of the same disease spectrum [6–8]. Janus kinase (JAK) inhibitors have been recently reported as promising treatment option for type I interferonopathies [9–11]. However, whether JAK inhibition also ameliorates symptoms in patients with SGMRT is unknown. Here, we report the case of a child with SGMRT1, in whom treatment with the JAK inhibitor ruxolitinib led to sustained clinical improvement.

## Case presentation

**Fig. 1:**
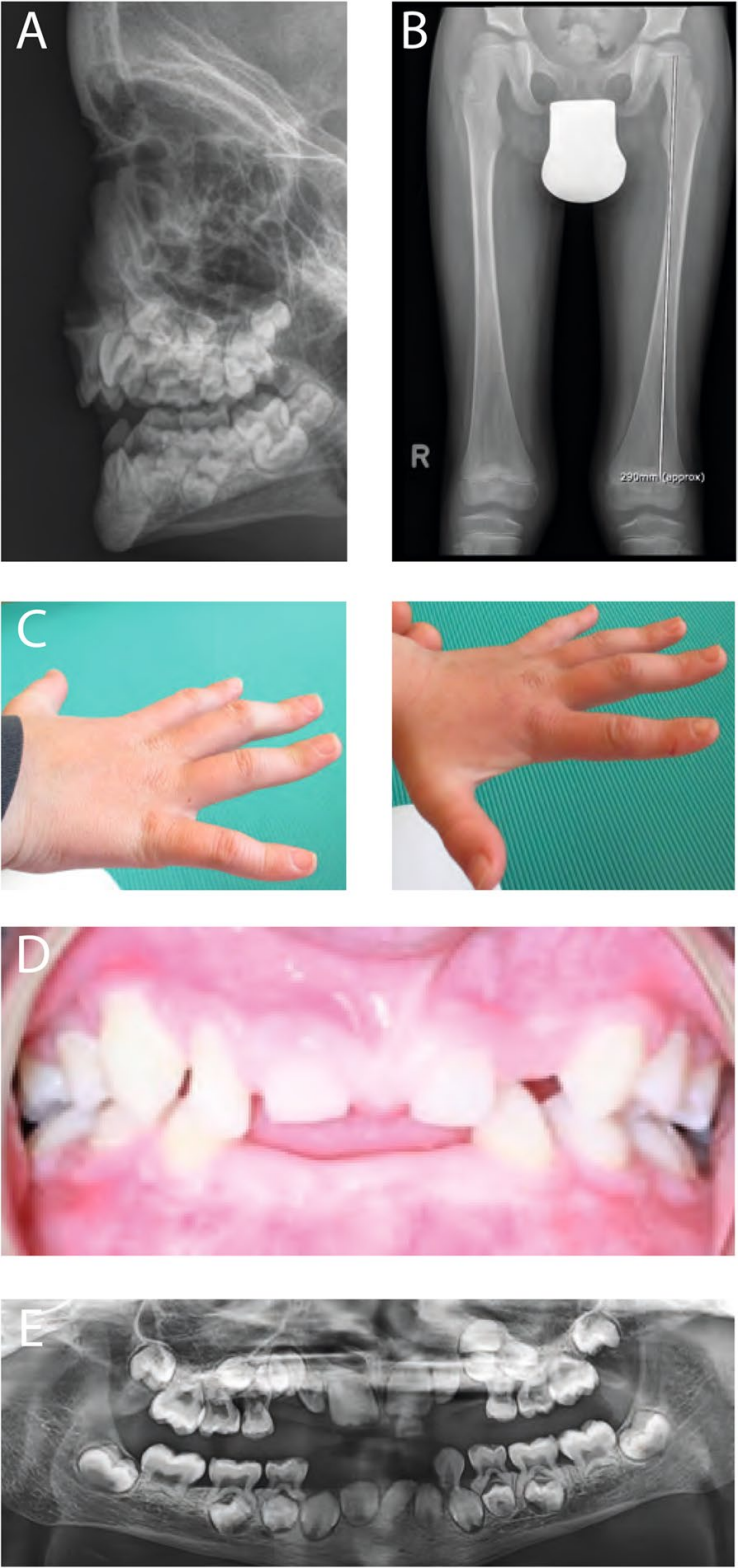
Clinical findings. **A** Lateral X-ray image of facial bones showing absence of nasal bone. **B** Anterior–posterior X-ray of the hip and femurs showing hypoplastic acetabular roofs, marked osteopenia of femoral bones and enlarged CCD angles. **C** Boutonniere deformity of hands. **D** Image of the teeth at the age of nine years, showing defective dentition with hypoplastic teeth. **E** Anterior–posterior x-ray of the denture showing aplasia of teeth no. 18, 28, 31, 38 and 48, as well as hypoplastic shortened roots of most of remaining teeth (courtesy of Dr. Zettel)

We report on a male patient who was born at term after an uncomplicated pregnancy to non-consanguineous parents. Birth weight, height and head circumference were within normal limits. The patient initially thrived well but was noted to reach developmental milestones later than expected. He was able to sit at 12 months of age, to stand at 18 months of age, and to walk unsupported at 27 months of age. At the age of four years, he presented with muscular weakness of the lower extremities. While he was able to walk on a flat surface, he had difficulties to climb up stairs or to squat. In addition, his growth stunted, and his length was below the 3rd percentile. During clinical examination, he showed a stiff gait with overextension of knee joints and a mild hyperlordosis. In contrast to the lower extremities, the patient exhibited a normal function of his upper extremities with a precise and well-controlled visual coordination of his hands. His speech and cognitive functions were within normal range. Neurophysiological examination revealed a discretely reduced motor nerve conduction speed (N. tibialis 39 m/s [*N* > 45], N. peroneus 43 m/s, [*N* > 45 m/s]) with unremarkable findings on repetitive nerve stimulation. His muscular mass was reduced with a normal muscle texture without any signs of inflammation on ultrasound and MR imaging. An MRI of the brain and spine showed normal findings. Endocrinological work-up for short stature revealed normal values for growth hormone, IGF-1 and IGFBP-III. His thyroid function test was normal and the inflammatory marker, C-reactive protein (CRP), was below 5 mg/ml. However, an X-ray of the hands showed osteopenia with a thinned cortex of the finger bones and a DEXA scan revealed reduced mineralization of bones (femur, Z-score -2.7SD; hip, Z-score -2.8SD; spine, Z-score -0.8SD). An X-ray of the skull revealed an absent nasal bone (Fig. 1A). The X-ray of the hip and femurs showed a pathological caput-collum-diaphyseal angle (Fig. 1B). The family declined further genetic testing at that point. The child was treated with physiotherapy and orthopedic support. Due to progressive pes equinus an Achilles tendon extension was performed.

**Fig. 2:**
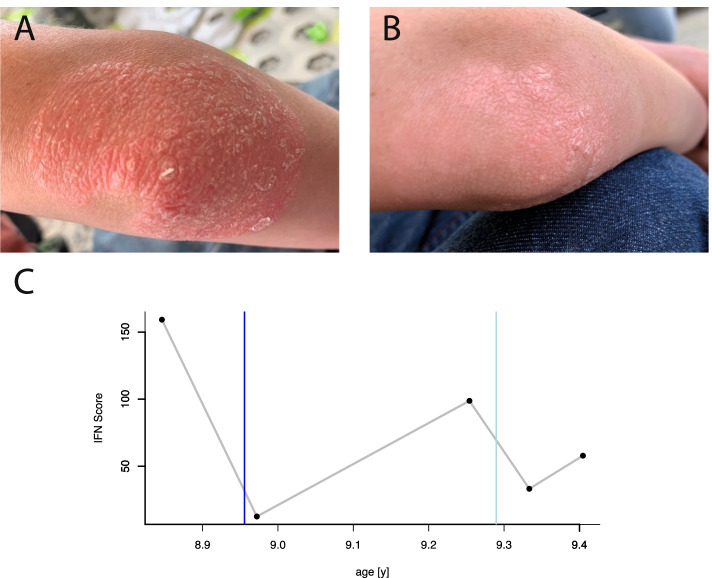
Effects of JAK inhibition on skin and interferon signature. **A** Right elbow before initiation of treatment with ruxolitinib, showing psoriatic lesion with hyperkeratosis and scaling. **B** Resolution of psoriatic skin lesion after one week of treatment. **C** Interferon signature before and during treatment with ruxolitinib. An IFN score < 12,49 defines the median of 10 healthy controls plus 2 SDs.The dark blue vertical line marks the start of the treatment with ruxolitinib. The light blue vertical line marks the time when the treatment was increased to the final dosage

**Fig. 3:**
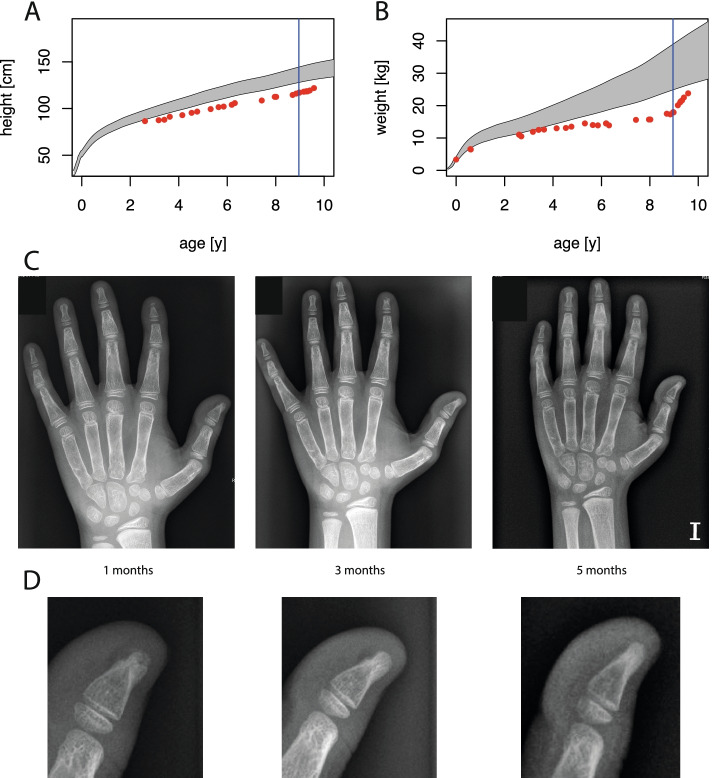
Effects of JAK inhibition on body weight and bone mineralization. **A** Growth chart showing development of height. The grey area indicates is the 5. to 95. percentiles for height of children in Europe. **B** Growth chart showing development of weight. The grey area indicates is the 5. to 95. percentiles for height of children in Europe. The dark blue vertical line at 9 years indicates start of treatment. **C** Series of palmar-dorsal hand X-rays of the left hand showing improved mineralization. The time points after initiation of treatment indicated below images. The scale bar on the lower right corner of panel **C** is 10 mm and applies to all images of panel **C**. **D** Enlarged images of thumb bones showing resolution of acro-osteolysis

In the following years, the patient exhibited normal cognitive development but his growth remained retarded and muscular weakness worsened. By the age of seven years, he could barely climb a stair without intense support. A boutonniere deformity of both hands was noted (Fig. 1C) and a hand X-ray showed acro-osteolysis (Fig. 3C, D), consistent with inflammatory bone destruction. At the age of eight years, the patient developed with severe psoriasis (Fig. 2A), unresponsive to steroid treatment. In addition, the patient exhibited failure of secondary dentition with aplasia of several teeth (18, 28, 31, 38 and 48) and hypoplastic roots of remaining teeth (Fig. 1D, E). A cardiologic examination, including echocardiography and electrocardiography, and an ophthalmologic exam were unremarkable.

The clinical findings of neuromuscular and inflammatory symptoms of bones and skin suggested a genetic etiology and eventually genetic testing was initiated. A trio exome analysis revealed a heterozygous de novo variant in the *IFIH1* gene (NM_022168.4: c.2465G > A; p.Arg822Gln) in the patient. This variant has been previously reported in patients with SGMRT1 [3], confirming the diagnosis of SGMRT1. Although the inheritance pattern of SGMRT1 is autosomal dominant, both healthy parents did not carry the disease-causing variant, p.Arg822Gln, indicating that it had occurred de novo. In addition, there was no evidence for a mosaic in the NGS data.

The IFN signature in blood was measured as previously described [12]. In line with constitutive type I IFN activation, peripheral blood mononuclear cells of the patient exhibited a strong interferon signature (an IFN score above 12.49), as shown by up-regulation of IFN-stimulated genes (Fig. 2C). Based on the genetic and laboratory findings and in view of the refractory skin disease, the decision was made to treat the child with ruxolitinib, a JAK1/2 inhibitor, which blocks signaling at the IFN-α/β receptor. Following oral administration of 0.5 mg/kg ruxolitinib per day, the child experienced significant improvement of psoriatic skin lesions that was already visible after three days of treatment and over the course resulted in complete resolution of cutaneous inflammation (Fig. 2B). In addition, the interferon signature was markedly reduced during treatment (Fig. 2C). Six weeks after treatment was started, the child showed an increase in body weight and length (Fig. 3A, B). A hand X-ray revealed a significant increase of bone mineralization of fingers (Fig. 3C). Remarkably, after five months of treatment, the acro-osteolysis of the thumb was completely resolved (Fig. 3D). The patient also experienced improvement of muscle weakness and his gross motor function classification system (GMFCS) score [13] improved from GMFCS level two (ambulatory with assistance) to GMFCS level one (independently mobile).

While the patient was on ruxolitinib, he experienced mild upper respiratory symptoms and elevated temperature for two days, followed by quick recovery. As the patient's sister had been tested SARS-CoV-2-positive, a COVID-19 infection was suspected. Serologic testing confirmed sero-conversation both anti- spike antigen IgG and IgM. Three months after initiation of ruxolitinib, the dosage was slightly increased to a final maintenance dose of 0.75 mg/kg per day. The treatment with ruxolitinib was well tolerated without any side effects. Repeated measurements of blood count and biochemistry did not show any changes and the patient felt very well.

## Discussion and conclusions

In 1973, Singleton and Merten and shortly later, in 1976, Gay and Kuhn reported four patients with dental dysplasia, osteoporosis, widened medullary cavities of hand bones and calcification of the thoracic aorta [14, 15]. In addition, some of the patients presented with muscle weakness as well as psoriasiform skin lesions. Feigenbaum et al. noted dominant inheritance with significant phenotypic variability of SGMRT even within families and summarized the core manifestations to include progressive aortic calcification, dental anomalies, osteopenia and acro-osteolysis and to a lesser extent, glaucoma, psoriasis, muscle weakness, and joint laxity [16]. In 2015, Rutsch et al. identified a p.Arg822Gln substitution in IFIH1 as the cause of SGMRT1 in three unrelated families and demonstrated by functional analysis that the mutation exerts a gain-of-function, resulting in a heightened inflammatory state due to overproduction of type I IFN [3]. Activating mutations in *IFIH1* were subsequently shown to underlie AGS7, an early-onset inflammatory leukencephalopathy characterized by basal ganglia calcification and constitutive type I IFN activation [2]. Notably, the p.Arg822Gln mutation initially identified in SGMRT1 was also observed in a patient presenting with typical clinical features of AGS, suggesting that SGMRT1 and AGS7 due to *IFIH1* gain-of-function mutations are part of the same disease spectrum [8].

The patient described here exhibited many of the typical features of SGMRT1, including dental anomalies, osteopenia, acro-osteolysis, psoriasis and muscle weakness, yet lacked the core feature of aortic calcification. Given that most patients with SGMRT1 develop aortic calcification early in childhood [17], the lack of this feature was unexpected. However, a patient with SGMRT1 without cardiac involvement carrying a different variant in the *IFIH1* gene (p.Leu329Pro) has recently been reported [18]. Nonetheless, due to the high cardiovascular risk we do monitor the patient regularly by echocardiography.

After establishing the diagnosis of SGMRT1 by genetic testing, we confirmed constitutive type I IFN activation in the patient by demonstrating up-regulation of IFN-stimulated genes in blood. Given the progressive disease course, in particular due to refractory skin inflammation, this led us to consider treatment with the JAK inhibitor ruxolinitib. While JAK inhibition had been shown to ameliorate symptoms in patients with type I interferonopathies, such as STING-associated vasculopathy, CANDLE syndrome or AGS [9–11], there have been no reports about targeted treatment approaches in SGMRT1 so far. Based on the assumption that uncontrolled type I IFN signaling was driving the inflammatory symptoms in our patient, we initiated treatment with ruxolitinib at 0.5 mg/kg bodyweight to inhibit type I IFN signaling. The patient responded with significant improvement that was visible within the first weeks of administration of the drug. Thus, psoriatic lesions vanished within days, the muscle weakness and bone mineralization improved, the patient showed a significant weight gain. Clinical improvement was accompanied by a marked reduction of the interferon signature in blood, indicating that inhibition of overactive type I IFN signaling was therapeutically effective.

In summary, this case report demonstrates that targeting of uncontrolled type I IFN activation by JAK inhibition is of therapeutic benefit in patients with SGMRT1 and highlights the role of precision medicine in the treatment of rare diseases in children. Moreover, our findings also suggest a hitherto unappreciated role of the interferon signaling pathway in bone metabolism.

## Data Availability

Not applicable.

## Abbreviations

CCD angle: Caput-collum-diaphyseal angle
DEXA scan: Dual energy X-ray absorptiometry
IFIH1: Interferon-induced with helicase C domain 1
IFN: Interferon
ISG: Interferon-stimulated gene
JAK: Janus kinase
MDA5: Melanoma differentiation-associated protein 5
RIG-I: Retinoic acid-inducible gene 1
RLR: RIG-I-like receptor
SGMRT1: Singleton-Merten syndrome 1
